# Case report: Allogeneic stem cell transplantation for type B insulin resistance

**DOI:** 10.3389/fmed.2023.1200037

**Published:** 2023-08-29

**Authors:** Thomas Ebert, Gerhard Behre, Lorenz Weidhase, Vladan Vucinic, Cornelia Gewert, Robert K. Semple, Michael Stumvoll, Anke Tönjes

**Affiliations:** ^1^Medical Department III—Endocrinology, Nephrology, Rheumatology, University of Leipzig Medical Center, Leipzig, Germany; ^2^University of Leipzig Medical Center, Medical Clinic and Policlinic 1, Hematology, Cellular Therapy and Hemostaseology, Leipzig, Germany; ^3^Clinic for Internal Medicine I, Community Hospital Dessau, Dessau, Germany; ^4^University of Leipzig Medical Center, Medical Intensive Care Unit, Leipzig, Germany; ^5^Metabolic Research Laboratories, Wellcome Trust-Medical Research Council Institute of Metabolic Science, University of Cambridge, Cambridge, United Kingdom; ^6^Cambridge Biomedical Research Centre, National Institute for Health Research, Cambridge, United Kingdom; ^7^Centre for Cardiovascular Science, Queen's Medical Research Institute, University of Edinburgh, Edinburgh, United Kingdom

**Keywords:** allogeneic peripheral blood stem cell transplantation, case report, rescue therapy, type B insulin resistance, Wiskott-Aldrich syndrome, diabetes

## Abstract

Type B insulin resistance (TBIR) is a rare, often fulminant form of insulin resistance caused by autoantibodies against the insulin receptor. If left untreated, its mortality is high. Various immunosuppressive regimens have shown efficacy, but treatment effects are variable and time-delayed, and drug-induced complications may arise. We report a patient with TBIR arising as a complication of Wiskott–Aldrich syndrome. Stable remission of TBIR was achieved through allogeneic peripheral blood stem cell transplantation (PBSCT) over a follow-up period of more than 1.5 years. We thus demonstrate that PBSCT can be considered a treatment option in TBIR where conventional immunosuppressive therapy is ineffective or contraindicated.

## Introduction

Type B insulin resistance (TBIR) is a rare and extreme form of insulin resistance described first in the 1970's (1, 2). If left untreated, mortality rates are high, often due to the combined effects of severe metabolic derangement and the complications of associated autoimmune diseases (3). TBIR is caused by autoantibodies against the insulin receptor (IR), with metabolic consequences determined by the titer and affinity of the autoantibody. In cellular studies and animal models, low concentrations of antibodies crosslink and stimulate IR (4, 5), but higher concentrations lead to sustained downregulation of the receptors and thus cellular insulin resistance (5). This is repeated clinically, with low antibody titers sometimes causing hypoglycemia, while higher titers lead to fulminant insulin resistance (6). Both states may be seen at different times during the natural history of the condition in the same patient as antibody titers and/or affinity fluctuate. Many immunosuppressive treatments for TBIR have been reported (7–9), encompassing different combinations of plasmapheresis, potent glucocorticoids, cyclophosphamide, cyclosporin A, azathioprine, and rituximab (10, 11). The most extensive evidence to date is related to a combination of rituximab, high-dose pulsed steroids, and cyclophosphamide (12). However, treatment of TBIR remains challenging, and treatment effects are time-delayed. Complications may arise from immunosuppression, while associated disorders may constrain therapeutic choices.

We present a case of TBIR in a 26-year-old European man with underlying Wiskott–Aldrich syndrome (WAS), a rare X-linked disorder caused by mutations in the *WAS* gene (13). WAS often features thrombocytopenia and susceptibility to infections, as well as a heterogeneous constellation of other complications, including autoimmune disorders, eczema, and cancer (13). After the development of hyperglycemia at the age of 24 years, sustained severe hyperglycemia and prolonged ketoacidosis developed, leading to the diagnosis of TBIR. Standard treatments did not improve glucose homeostasis, and WAS-related infectious and hematological complications arose, leading to allogeneic peripheral blood stem cell transplantation (PBSCT) as a rescue therapy. This led to the clearance of anti-insulin receptor autoantibodies and the normalization of glycemia over a follow-up period of 1.5 years to date.

## Methods

The patient was admitted to Leipzig University Hospital in September 2017 at the age of 26 years. Longitudinal evaluations included clinical, anthropometric, biological, and metabolic assessments. A continuous glucose monitoring (CGM) system (FreeStyle^®^ Libre™ system, Abbott Diabetes Care, Alameda, CA) was employed, with outpatient monitoring undertaken over 2–4 weeks every 3 months and analyzed using the FreeStyle Libre desktop software (Abbott Diabetes Care, Alameda, CA). Intravenous insulin dosage was calculated each day until remission of hyperglycemia. Written informed consent was given for publication.

### Biochemical analyses

For all time points, blood and urine samples were collected after an overnight fast. Blood was immediately centrifuged and stored at −80°C until analyses were performed. All routine clinical analytes were measured by standard laboratory methods in a certified laboratory at Leipzig University Hospital.

### Immunoprecipitation assay of IR autoantibodies

IR autoantibodies were determined by a previously described semi-quantitative immunoprecipitation assay at the Metabolic Research Laboratories of the University of Cambridge, UK (14). In brief, serum was incubated with a preparation of human insulin receptor prior to the pulldown of IgG and immunoblotting for the insulin receptor. Immunoblots presented feature single samples from each time point throughout the treatment period.

### Allogeneic PBSCT protocol

Repeated unrelated donor searches for PBSCT were undertaken after the failure of medical TBIR therapy, eventually identifying an eligible human leukocyte antigen (HLA)-A-antigen-mismatch (9/10) unrelated donor. Despite PBSCT being the standard of care for WAS and its complications (15), the treatment strategy was approved by an internal institutional review board. Afterward, an allogeneic, HLA-A-antigen-mismatch (9/10) PBSCT was performed after reduced-intensity conditioning using Fludarabine (30 mg/m^2^/day for 3 days) and total body irradiation with 2 Gy as previously described (16). Total 7.38 × 10^6^ CD34^+^ cells/kg body weight and 2.1 × 10^8^ CD3^+^ cells/kg body weight were transplanted. Cyclosporine A and mycophenolate mofetil were used as graft-vs.-host disease (GVHD) prophylaxis regimens. Cyclosporine A was started 1 day before PBSCT and was adjusted to a target blood level of 200 ng/ml. Mycophenolate mofetil was administered starting from day 0, 8 h after PBSCT, at a dosage of 1,000 mg three times a day. In the absence of apparent acute GvHD, the prophylaxis with mycophenolate mofetil was reduced by 500 mg every 2 weeks starting from day 40 after PBSCT.

The PBSCT treatment was performed in an inpatient setting on a transplant ward in accordance with the international guidelines for preventing infectious complications (17). The isolation was performed until the regeneration of neutrophil granulocytes (>500/μl) in a single room with high-efficiency particulate air filtration.

## Results

In a 26-year-old European man, WAS (13) had been clinically diagnosed at the age of 2 years, with a causative *WAS* mutation (NM_000377.2; c.1052delC) identified later. The predominant clinical manifestations of WAS were autoimmune hemolytic anemia and thrombocytopenia, in accordance with the autoimmune WAS phenotype previously described for this mutation (18, 19). The patient denied a history of consanguinity in his family. Furthermore, no other family member was affected by WAS. During childhood, allogeneic hematopoietic stem cell transplantation (allo-HCT) was considered. However, the patient had no siblings, and repeated searches (1994, 2007) could not identify an HLA-matched unrelated stem-cell donor. Baseline immunological and hematological markers prior to PBSCT are shown in Table 1.

**Table 1:** Baseline immunological and hematological investigations include lymphocyte subset counts, immunoglobulin (Ig) levels, and serum protein electrophoresis.

	**Absolute**	**Relative (%)**	**Day according to PBSCT**	**Clinical event**
**Serum protein electrophoresis**				Admission to hospital
Albumin (g/L)	36.0	57.4	−165	
Alpha-1 globulin (g/L)	3.8	6.0	−165	
Alpha-2 globulin (g/L)	6.3	10.1	−165	
Beta-1 globulin (g/L)	3.4	5.5	−165	
Beta-2 globulin (g/L)	1.9	3.1	−165	
Gamma globulin (g/L)	11.2	17.9	−165	
**Immunoglobulin levels**				Admission to hospital
IgA (g/L)	1.23	-	−65	
IgG (g/L)	11.47	-	−165	
IgM (g/L)	0.64	-	−165	
IgA (g/L)	1.20	-	−111	After end of plasmapheresis/first rituximab course
IgG (g/L)	6.97	-	−111	
IgG1 (g/L)	3.978	-	−111	
IgG2 (g/L)	2.455	-	−111	
IgG3 (g/L)	0.330	-	−111	
IgG4 (g/L)	0.507	-	−111	
IgM (g/L)	0.23	-	−111	
**Lymphocyte subset counts**				Prior to PBSCT
Leucocytes (× 10^9^/L)	2.4	-	−54	
Lymphocytes (× 10^9^/L)	0.192	8	−54	
T cells (CD3+) (× 10^9^/L)	0.149	77.5	−54	
B cells (CD19+) (× 10^9^/L)	0	0	−54	
NK cells (CD3-/CD16/56+) (× 10^9^/L)	0.043	22.5	−54	
Activated T cells (CD3+/HLA-DR+) (× 10^9^/L)	0.075	39	−54	
TH/I cells (CD3+/CD4+) (× 10^9^/L)	0.065	34	−54	
TC/S cells (CD3+/CD8+) (× 10^9^/L)	0.081	42	−54	
CD4/CD8-ratio	-	0.81	−54	

Absolute and/or relative levels are given, as well as the day of blood sampling according to PBSCT and clinical events. NK, natural killer; PBSCT, peripheral blood stem cell transplantation.

Hyperglycemia developed at the age of 24 years. Given a body mass index of 34 kg/m^2^, he was diagnosed with type 2 diabetes and treated with different glucose-lowering agents, including insulin. Initiation of antidiabetic treatment was followed by a 50 kg weight loss, with persisting hyperglycemia despite regular insulin dose adjustments. Two years after diabetes was diagnosed, the patient was referred to our tertiary hospital with persistent hyperglycemia, ketoacidosis, and thrombocytopenia.

**Figure 1:**
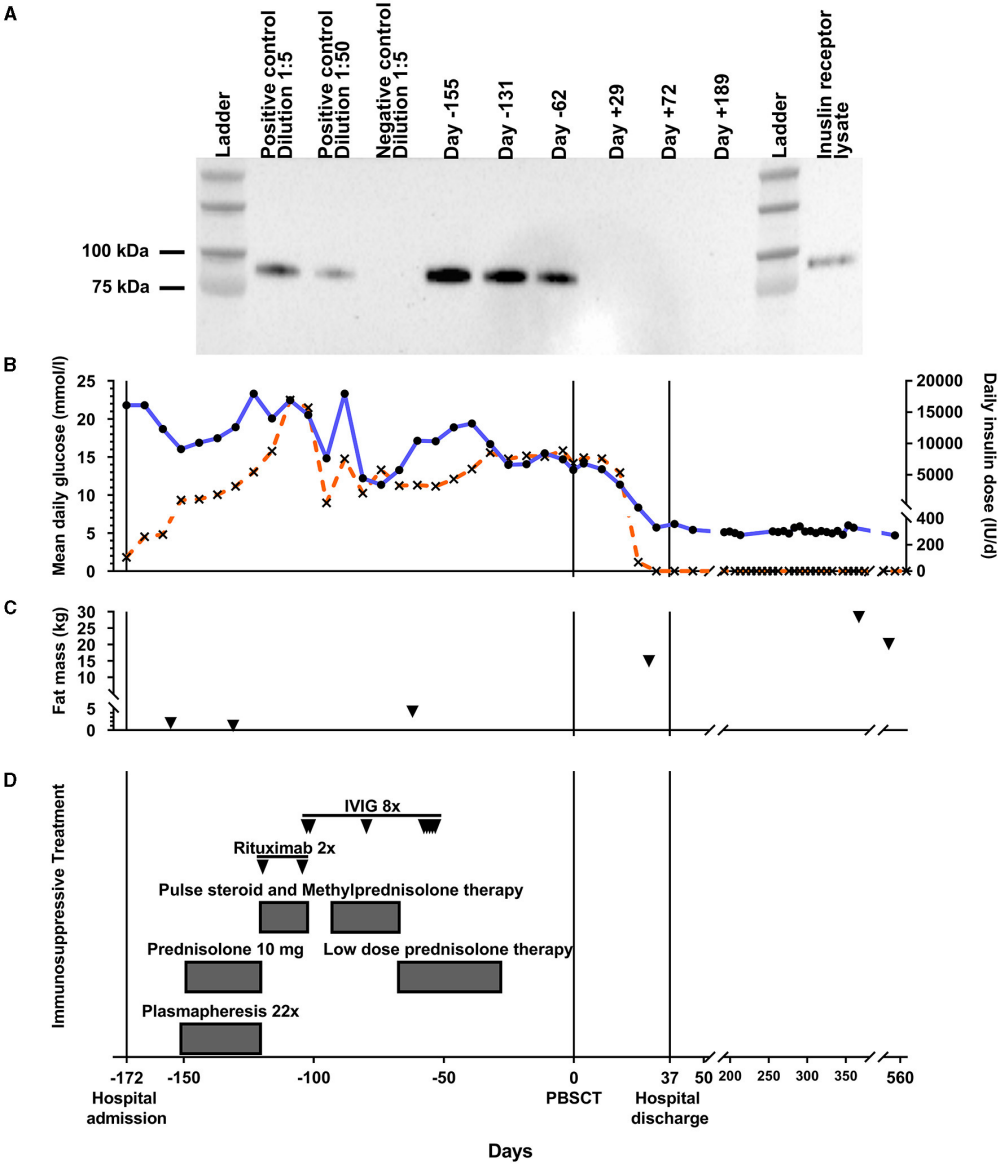
Glucose homeostasis and TBIR treatment approaches over time. during conventional therapy (day -172 to -1), after allogeneic PBSCT (days 0–37), and during follow-up (days 38–553). **(A)** Longitudinal results of an anti-insulin receptor (IR) autoantibody assay (20-min exposure) for our patient during conservative and peripheral blood stem cell transplantation (PBSCT) treatment. The blot was made from a single batch of loading samples from each time point throughout the treatment period. A positive control from a different patient with proven TBIR and different dilutions, as well as a negative control, are included. Lane 12 shows a cell lysate of CHO cells stably expressing human IR and run without prior immunoprecipitation. **(B)** Mean daily glucose levels (left scale and black circles/blue line) and daily intravenous insulin dose (right scale and black crosses/orange line) plotted over time during conventional therapy, after allogeneic PBSCT, and during follow-up. Mean daily glucose levels and insulin doses were averaged and are depicted per week. **(C)** Fat mass (black triangles) as assessed by bioelectrical impedance analysis longitudinally throughout the treatment period. Conventional TBIR treatment approaches are depicted in **(D)**. IVIG, intravenous immunoglobulin therapy; Pos/Neg con., positive/negative control; TBIR, type B insulin resistance.

Negative islet autoantibodies were confirmed, and severe hyperglycemia proved refractory to massively increased intravenous insulin therapy (maximum dosage of 53,000 units/day) (Figure 1B). A clinical diagnosis of a WAS-related autoimmune defect in the insulin signaling cascade was made, and the diagnosis of TBIR was confirmed by immunoprecipitation assay, which showed strongly positive anti-IR autoantibodies (Figure 1A). High serum adiponectin (54.0 mg/L) strongly supported the diagnosis (20).

Immediately after TBIR diagnosis, plasmapheresis with low-dose prednisolone was started (Figure 1D). No clinically meaningful metabolic improvement was discernible, and strongly positive anti-IR antibodies persisted in the immunoprecipitation assay (Figure 1A). Pulsed intravenous corticosteroid therapy was then tried in combination with one cycle (two doses) of rituximab. WAS-related thrombocytopenia did not allow standard cyclophosphamide treatment (12). Instead, intravenous immunoglobulin was administered. After 3 months of conservative TBIR therapies, hyperglycemia and practically absent adipose tissue (Figure 1C and Table 2) were unchanged, indicating a failure of conventional treatment approaches. Immunosuppressive treatment resulted in severe opportunistic infections complicated by septic shock, acute respiratory distress syndrome, right ventricular failure, and disseminated intravascular coagulation, requiring prolonged intensive care unit stay and complex treatment. The repeated donor search attempt identified an HLA-A-antigen-mismatch (9/10) unrelated donor. The hematopoietic cell transplantation-specific comorbidity index (HCT-CI) (21) indicated a high-risk patient, ineligible for myeloablative, but qualifying for non-myeloablative conditioning regimens (HCT-CI=7).

**Table 2:** Longitudinal anthropometric data and glucose homeostasis of the patient with type B insulin resistance due to Wiskott-Aldrich syndrome throughout the treatment period.

**Day according to PBSCT**	**−155**	**−131**	**-62**	**+29**	**+189**	**+367**	**+553**
Clinical event	Diagnosis	Plasmapheresis	Pre-PBSCT	Diabetes remission	0.5-year follow-up	1-year follow-up	1.5-year follow-up
Body weight (kg)	59.0	77.5	70.7	72.6	81.9	97.5	110.8
BMI (kg/m^2^)	19.0	25.0	22.8	23.4	26.4	31.5	35.8
FFM (kg) [% body weight]	57.4 [97.2]	76.5 [98.7]	66.4 [93.9]	57.7 [79.5]	62 [75.7]	69.1 [70.9]	90.6 [81.8]
Fat mass (kg) [% body weight]	1.6 [2.7]	1.0 [1.3]	4.3 [6.0]	14.9 [20.5]	19.9 [24.3]	28.4 [29.1]	20.2 [18.2]
Mean 24 h glucose (mmol/L)	18.7	18.9	17.1	5.7	5.1	5.7	4.7
Daily i.v. insulin dosage (IU/day)	278	3,157	3,337	0	0	0	0
HbA1c (% [mmol/mol])	6.4 [47]	-	6.3 [46]	4.7 [28]	5.3 [34]	5.2 [33]	5.3 [35]

Mean daily glucose and insulin levels were averaged per week. The glucose level at day +553 was derived from one fasting glucose test. HbA1c could not be used as a marker of glycemic control due to WAS-related hemolytic anemia. BMI, body mass index; FFM, fat-free mass; HbA1c, glycated hemoglobin A1c; i.v., intravenous; PBSCT, peripheral blood stem cell transplantation.

An allogeneic, unrelated donor, HLA-A-antigen-mismatch PBSCT was undertaken 6 months after referral to our hospital on 20 March 2018. The engraftment of white blood cells (absolute neutrophil count >500/μl) and platelets (>50,000/μl) occurred 19 and 20 days after PBSCT, respectively. No signs of acute GvHD were detected. Under the tapering of immunosuppression, the patient developed signs of a limited chronic skin GvHD. Therefore, the Cyclosporin A therapy was not discontinued.

From 19 days after the transplant, we observed improved glucose profiles and could quickly wean intravenous insulin, stopping entirely at day 26 (Figure 1B). At day 29 after PBSCT, glycemia was in the target range, and anthropometric markers improved (Figure 1C and Table 2). Anti-IR autoantibodies were confirmed to be undetectable (Figure 1A). The patient was discharged from the hospital on day 37 after PBSCT without antidiabetic treatment. Over 1.5 years of follow-up, glucose concentrations have remained in the target range without antidiabetic treatment, and anti-IR autoantibodies were still undetectable 6 months after PBSCT (Figures 1A, B).

Approximately 2 years after PBSCT, renal function deteriorated to Kidney Disease: Improving Global Outcomes (KDIGO) stage G3bA3. The donor chimerism was 100%, and a kidney biopsy excluded the relapse of the disease but revealed a phospholipase A2 receptor-negative membranous nephropathy. Immunosuppressive treatment with methylprednisolone and an increase in serum level of cyclosporine A were initiated, resulting in stabilized renal function.

### Body composition over the treatment period

Adipose tissue was practically absent from the time of specialist referral to PBSCT, consistent with a prolonged catabolic state and the sustained ketoacidosis observed (Figure 1C and Table 2). Prior to PBSCT, body weight and BMI increased after relieving ketoacidosis, most likely through rehydration (Table 2); however, fat mass and other anthropometric markers improved only after PBSCT (Figure 1C and Table 2).

## Discussion

We describe the first case of TBIR due to WAS and the first-in-human use of PBSCT to induce sustained remission of TBIR over a follow-up period of >1.5 years. WAS-related complications, as well as the lack of metabolic response to immunosuppressive therapy, precluded standardized immunosuppressive treatment protocols for TBIR (12), thereby leading to the novel use of PBSCT to clear pathogenic antibodies (22).

Patients with WAS frequently suffer from a wide range of autoimmune disorders, such as autoimmune cytopenia, and other diverse autoimmune diseases (13). Pathophysiologically, both T cells (e.g., natural regulatory T cells) and B cells (e.g., regulatory B cells) are dysfunctional in WAS, thereby mediating an increased burden of autoimmune disorders in affected individuals [reviewed in Candotti (13)] It is, therefore, tempting to speculate that autoantibody-mediated TBIR in our patient was based on the significantly increased susceptibility to autoimmune diseases in WAS.

PBSCT quickly restored glucose to the target range and permitted the withdrawal of antidiabetic treatment. The metabolic benefits of PBSCT were further evidenced by bioelectrical impedance analysis. In more detail, the fat mass did not increase in a clinically relevant manner despite immunomodulating treatment prior to PBSCT (Table 2). Only at the very last measurement prior to PBSCT, a small increase in the fat mass was observed, most likely, through rehydration (Table 2). In contrast, after PBSCT and relief of TBIR, the fat mass consistently increased, indicating recovered and physiological insulin signaling.

The action of PBSCT to clear anti-IR autoantibodies was proven at 29 days after PBSCT and sustained over 6 months of follow-up. The exact pathophysiological mechanisms by which stem cell transplantation can exert beneficial effects on autoimmune diseases still need to be defined in more detail. During the last few years, clinical experience and immunological knowledge on stem cell transplantation for autoimmune diseases have expanded predominantly for autologous approaches (23), whereas there is only limited data for allogeneic stem cell transplantation (24). Hypothetically, an exchanged, donor-derived lymphohematopoietic system after allogeneic stem cell transplantation could induce an immunological tolerance/reset with a complete and sustained remission of autoantibody-mediated TBIR. It should be noted that hematopoietic stem cell transplantation as a treatment option for autoimmune diseases may only be considered on an individual-case basis for rare indications (22). While PBSCT is an established treatment approach for WAS, PBSCT as a treatment option for autoimmune TBIR has not been investigated so far.

Interestingly, allogeneic bone marrow transplantation has also been used to treat the autoimmune destruction of pancreatic beta cells in immunodysregulation, polyendocrinopathy, enteropathy, and X-linked syndrome (25). Here, autoantibodies are significantly reduced or eliminated at 1 month after transplantation (25), which is comparable to our patient with TBIR, supporting the hypothesis that PBSCT can be a treatment option for autoantibody-mediated autoimmune diseases in limited individual cases, such as TBIR.

Some limitations of the current case report need to be pointed out. First, our data are based on only one case report. Thus, future studies need to elucidate the pathophysiological mechanisms by which PBSCT alleviates autoimmune TBIR. Second, we did not assess the protein expression of WAS protein, which could have verified the pathogenic effect of the causative WAS mutation in our patient. On the other hand, we confirm the metabolic effects of PBSCT, i.e., the remission of hyperglycemia without antidiabetic treatment, through undetectable anti-IR autoantibodies longitudinally up to 6 months after PBSCT. Furthermore, we have used a state-of-the-art CGM system to precisely collect glucose data every 5 min during inpatient and outpatient care.

In conclusion, it appears feasible to consider PBSCT as a treatment option for autoimmune TBIR in case of contraindications or other underlying factors prohibiting conventional TBIR therapy.

## Data availability statement

The original contributions presented in the study are included in the article/supplementary material, further inquiries can be directed to the corresponding author.

## Ethics statement

Written informed consent was obtained from the individual(s) for the publication of any potentially identifiable images or data included in this article.

## Author contributions

TE, GB, LW, VV, and AT treated the patient, collected the patient's material, and provided and interpreted the clinical data. CG and RS performed the immunoprecipitation assay of the insulin receptor autoantibodies. TE, RS, and AT contributed to the writing of the manuscript. All authors contributed to the interpretation of the results and editing of the manuscript.
